# CXCR4: A Promising Novel Strategy for Lung Cancer Treatment

**DOI:** 10.3390/biom16020188

**Published:** 2026-01-26

**Authors:** Mengting Liao, Jianmin Wu, Tengkun Dai, Guiyan Liu, Jiayi Zhang, Yiling Zhu, Lin Xu, Juanjuan Zhao

**Affiliations:** 1 Department of Immunology, Zunyi Medical University, Zunyi 563000, China; 2 Key Laboratory for Cancer Prevention and Treatment of Guizhou Province, Zunyi 563000, China

**Keywords:** lung cancer, *CXCR4*, expression patterns, mechanism of action, therapeutic target

## Abstract

Lung cancer remains a major public health challenge due to high incidence and mortality. The chemokine receptor *CXCR4* and its ligand *CXCL12* (SDF-1) constitute a critical axis in tumor biology, influencing tumor cell proliferation, invasion, angiogenesis, and immune evasion. Aberrant *CXCR4* expression is frequently observed in lung cancer and is closely associated with adverse prognosis, enhanced metastatic potential, and therapeutic resistance. Mechanistically, *CXCR4* activates signaling pathways including *PI3K*/*AKT*, *MAPK*/*ERK*, *JAK*/*STAT*, and *FAK*/*Src*, promoting epithelial–mesenchymal transition, stemness, and survival. The *CXCL12*/*CXCR4* axis also orchestrates interactions with the tumor microenvironment, facilitating chemotaxis toward *CXCL12*-rich niches (e.g., bone marrow and brain) and modulating anti-tumor immunity via regulatory cells. Regulation of *CXCR4* occurs at transcriptional, epigenetic, and post-transcriptional levels, with modulation by hypoxia, inflammatory signals, microRNAs, and post-translational modifications. Clinically, high *CXCR4* expression correlates with metastasis, poor prognosis, and reduced response to certain therapies, underscoring its potential as a prognostic biomarker and therapeutic target. Therapeutic strategies targeting *CXCR4* include small-molecule antagonists (e.g., AMD3100/plerixafor; balixafortide), anti-*CXCR4* antibodies, and *CXCL12* decoys, as well as imaging probes for patient selection and response monitoring (e.g., 68Ga-pentixafor PET). Preclinical and early clinical studies suggest that *CXCR4* blockade can impair tumor growth, limit metastatic spread, and enhance chemotherapy and immunotherapy efficacy, although hematopoietic side effects and infection risk necessitate careful therapeutic design. This review synthesizes the molecular features, regulatory networks, and translational potential of *CXCR4* in lung cancer and discusses future directions for precision therapy and biomarker-guided intervention.

## 1. Introduction

Lung cancer remains a major global health burden, with incidence and mortality rising worldwide since 2011. According to the 2022 Global Cancer statistics, it was the most prevalent cancer, accounting for approximately 2.5 million new cases (about 12.4% of all cancers) and the leading cause of cancer-related death, responsible for about 1.8 million deaths (18.7%) [1]. Lung cancer is primarily categorized into two major types: non-small cell lung cancer (NSCLC) and small cell lung cancer (SCLC). NSCLC accounts for approximately 80% to 85% of cases, including histological subtypes such as adenocarcinoma and squamous cell carcinoma, while SCLC constitutes the remaining portion [2]. As the most common type of lung cancer, NSCLC exhibits high incidence and mortality rates. Current treatment options primarily include surgical resection, chemotherapy, targeted therapy, and radiation therapy. Despite recent advancements in lung cancer research, the overall prognosis for patients remains grim due to the complex and poorly understood mechanisms of the disease, leading to low five-year survival rates. Consequently, there is an urgent need to explore more effective treatment strategies and identify new clinical treatment approaches.

Recent advancements in biotechnology and interdisciplinary research have led to breakthrough opportunities in the field of lung cancer treatment [3,4,5]. Notably, the advent of gene therapy offers significant potential for the identification and development of novel therapeutic targets [6]. In this context, the C-X-C chemokine receptor 4 (*CXCR4*) and its ligand *CXCL12* have garnered widespread attention. Research indicates that *CXCR4* plays a central role in tumor occurrence, development, and metastasis. It regulates cancer cell proliferation, survival, angiogenesis, and invasive capacity, while also influencing the formation and maintenance of the tumor microenvironment. Moreover, *CXCR4* participates in the dynamic balance of the immune system by regulating the migration and hematopoietic function of immune cells. Therefore, abnormal overexpression of *CXCR4* is closely associated with the occurrence, development, and prognosis of various tumors, including lung cancer, leukemia, breast cancer, prostate cancer, and multiple myeloma.

Given the critical role of *CXCR4* in lung cancer progression, researchers have conducted in-depth explorations into its potential as a therapeutic target. Targeting *CXCR4* through various intervention strategies is anticipated to provide new treatment options for lung cancer patients, thereby enhancing their survival rates and quality of life. This paper aims to systematically review the expression patterns and mechanisms of action of *CXCR4* in lung cancer, as well as summarize the current status and future prospects of its use as a therapeutic target. By doing so, it seeks to offer new perspectives and directions for the precision diagnosis and treatment of lung cancer.

## 2. Overview of CXCR4

The *CXCR4* gene, located on chromosome 2q22.1, is an important member of the G protein-coupled receptor (GPCR) family [7]. It features the characteristic seven transmembrane domain structure and is composed of 352 amino acid residues. This structure includes a highly conserved N-terminal extracellular domain, seven transmembrane α-helical regions (TM1–TM7), and an intracellular loop domain [8,9,10]. *CXCR4* is widely expressed in human tissues, particularly showing significant expression patterns in organs such as bone marrow, thymus, stomach, and lungs, highlighting its key critical regulatory role in various physiological and pathological processes [10].

At the molecular level, the N-terminal extracellular domain of *CXCR4* exhibits a high affinity for its ligand, stromal cell-derived factor 1 (SDF-1, also known as *CXCL12*). Notably, the third intracellular loop of *CXCR4* demonstrates a unique structure-function duality: it not only directly engages in ligand recognition and binding through specific amino acid residues but also facilitates G protein-coupled signal transduction via conformational changes. This process precisely regulates the intensity and duration of downstream signaling. During signal transduction, *CXCR4* relies on the conserved DRY motif (Asp-Arg-Tyr triplet) in the transmembrane region and requires the coordinated formation of specific spatial conformations among the seven transmembrane domains. Recent studies revealthat the tetrameric conformation of *CXCR4*, multi-ligand competitive binding, and oligomerization regulation provide new avenues for investigating the co-receptor functional domain and ligand-binding domain, thereby advancing the development of precision-targeted therapeutics in tumor metastasis and related fields [11,12,13].

*CXCR4* fulfills numerous roles, such as mediating cellular signal transduction by coupling with inhibitory G proteins to affect cell migration, adhesion, and gene transcription [14]. In hematopoietic stem cell homing, *CXCR4* interacts with *CXCL12* to regulate the migration of *CD34*^+^ hematopoietic stem cells within the bone marrow [15]. Additionally, *CXCR4* binds with high affinity to *CXCL12*, facilitating the migration of various cell types [16]. It also plays a crucial role in coordinating innate and adaptive immune responses by regulating leukocyte transport and distribution in peripheral tissues, promoting lymph node tissue formation, stabilizing immune synapses, and maintaining T cell activation [17]. In the context of tumor growth and metastasis, *CXCR4* is instrumental in processes such as tumor growth, invasion, angiogenesis, metastasis, and treatment resistance. Moreover, the *CXCL12*/*CXCR4* axis promotes angiogenesis, with signaling molecules stimulating *CXCL12*/*CXCR4* expression [18].

*CXCL12* (also known as SDF-1) is the sole ligand for *CXCR4*, and the *CXCR4*/*CXCL12* axis participates in numerous biological processes by activating pathways such as *PI3K*-*AKT*, *MAPK*/*ERK*, *JAK*/*STAT*, and others to regulate migration, invasion, and metastasis [19]. The axis is prominently expressed across multiple tumor types and plays a significant role in shaping the tumor microenvironment (TME) by modulating immune cell trafficking and function, thereby supporting tumor progression. Specifically, the *CXCR4*/*CXCL12* axis interacts with T cell and B cell receptors (TCR and BCR), in conjunction with *CD47*, to promote macrophage phagocytosis of tumor cells, an interaction referred to as immune gene surrender (IGS) [8]. Therefore, the *CXCR4*/*CXCL12* axis holds significant promise in tumor immunotherapy. The diversity of the *CXCR4*/*CXCL12* axis is reflected in the different subtypes of its ligand, *CXCL12*. Currently, seven *CXCL12* subtypes have been identified (α, β, γ, δ, ε, θ, and the predicted iso7), with α and β being the most extensively studied forms, consisting of 89 and 93 amino acids, respectively [20,21]. The γ subtype is highly expressed in organs with low vascularization, such as the heart and brain. Although it has lower efficacy, it exhibits the highest binding affinity for *CXCR4* and the longest duration of downstream effects [22,23].

In lung cancer, metastasis is the primary cause of poor prognosis and high recurrence rates, while epithelial–mesenchymal transition (EMT) is a key pathological factor in the early metastasis of primary lung cancer. Studies have shown that abnormalities in the *CXCL12*-*CXCR4* signaling axis play a significant role in tumor cell EMT, invasion, and chemotherapy resistance [24]. Beyond tumor cell–intrinsic effects, the axis participates in shaping immune cell infiltration and organ-specific colonization, acting as a guiding, driving, and targeting signal for metastatic spread.

**Figure 1:**
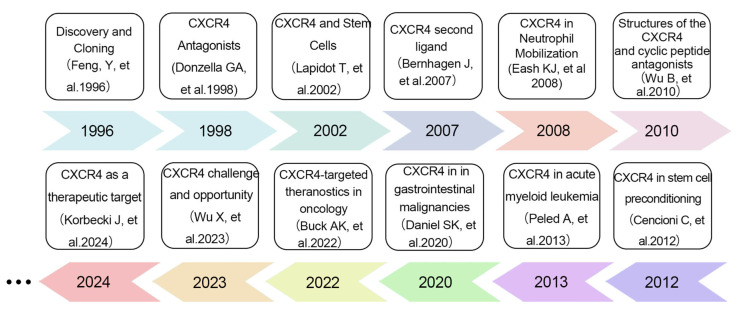
Overview of the Major Advances in Research on the Association Between *CXCR4* and Various Diseases [25,26,27,28,29,30,31,32,33,34,35,36]. 1996, Feng et al. cloned *CXCR4* and identified it as a key co-receptor for HIV-1 entry into T cells [25]; 1998, Donzella et al. demonstrated AMD3100 blocks HIV-1 via *CXCR4*, elucidating the first *CXCR4* antagonist mechanism [26]; 2002, Lapidot et al. clarified the *CXCR4/SDF-1* axis’s core role in human hematopoietic stem cell homing and post-transplant reconstitution via immunodeficient mouse models, providing critical clinical translation evidence [33]; 2007, Bernhagen J et al. identified MIF as a second endogenous ligand of *CXCR4* [27]; 2008, Eash KJ et al. confirmed *CXCR4*’s central role in neutrophil release from bone marrow [34]; 2010, Wu B et al. resolved the crystal structure of *CXCR4* in complex with a small-molecule antagonist [28]; 2012, Cencioni et al. revealed *CXCR4*’s role in stem cell pretreatment and cardiovascular repair [35]; 2013, Peled et al. explored *CXCR4*’s role in AML pathogenesis [29]; 2020, Daniel et al. reported the *CXCL12-CXCR4/CXCR7* axis mediates immune resistance in gastrointestinal malignancies [30]; 2022, Buck et al. reviewed *CXCR4*-targeted theranostic advances in oncology [31]; 2023, Wu et al. defined the *CXCL12/CXCR4* axis’s regulatory role in fibrotic diseases [36]; 2024, Korbecki et al. validated *CXCR4* as a therapeutic target in AML [32].

In summary, as a multifunctional GPCR, *CXCR4* exhibits widespread distribution and diverse functions that are important in both normal physiology and disease (Figure 1). Landmark studies over the past three decades have continuously deepened our understanding of its pleiotropic roles. In 1996, Feng et al. published a seminal work in Science that first cloned *CXCR4* and identified it as a key coreceptor mediating HIV-1 entry into T cells, laying the fundamental groundwork for subsequent functional explorations of this receptor [25]. Two years later, Donzella et al. reported in Nature Medicine that the small-molecule inhibitor AMD3100 blocks HIV-1 infection by targeting *CXCR4*, which was the first demonstration of the action mechanism of *CXCR4* antagonists and provided critical candidate molecules for anti-HIV drug development [26]. A pivotal breakthrough in deciphering *CXCR4* ligand interactions came in 2007, when Bernhagen J et al. identified macrophage migration inhibitory factor (*MIF*) as a second endogenous ligand for *CXCR4*, challenging the long-held dogma of exclusive *CXCL12*-*CXCR4* binding and revealing the receptor’s ligand diversity [27]. The molecular underpinnings of *CXCR4* function were further elucidated in 2010, when Wu B et al. resolved the first crystal structure of *CXCR4* in complex with a small-molecule antagonist, offering an essential structural blueprint for targeted drug design [28]. In the realm of oncology, *CXCR4* has steadily emerged as a promising therapeutic target, with accumulating evidence highlighting its involvement in tumor pathogenesis and progression across multiple malignancies. In 2013, Peled et al. demonstrated in Theranostics its critical role in the pathogenesis of acute myeloid leukemia (AML), clarifying its context-specific regulatory mechanisms in hematologic malignancies [29]. In 2019, Daniel et al. expanded this perspective in Seminars in Cancer Biology, revealing that the *CXCL12*-*CXCR4*/*CXCR7* signaling axis mediates immune resistance in gastrointestinal malignancies and providing novel insights for combining targeted therapy with immunotherapy [30]. Progress in clinical translation was summarized in 2022 by Buck et al. in European Journal of Nuclear Medicine and Molecular Imaging, who systematically reviewed advances in *CXCR4*-targeted theranostic strategies and accelerated the translation of basic research into clinical practice [31]. Most recently, in 2024, Korbecki et al. further validated the feasibility of *CXCR4* as a therapeutic target in AML in a study published in Leukemia, proposing optimized intervention strategies that offer updated support for precision oncology [32]. Against this backdrop of expanding insights into *CXCR4*-mediated oncogenic pathways across cancer types. In the context of lung cancer, the *CXCR4*/*CXCL12* axis holds considerable potential for understanding tumor occurrence, progression, and metastasis, offering valuable insights for future therapeutic strategies. In the context of lung cancer, the *CXCR4*/*CXCL12* axis holds considerable potential for understanding tumor occurrence, progression, and metastasis, offering valuable insights for future therapeutic strategies.

## 3. Molecular Regulation of CXCR4 Expression

*CXCR4* is a chemokine receptor widely expressed across various cell types, particularly in immune cells, hematopoietic stem cells, and multiple tumor cell types. It is notably overexpressed in diverse tumors, including lung cancer [23]. Beyond facilitating tumor cell migration and invasion, *CXCR4* also modulates tumor immune responses by impacting immune cell functions within the tumor microenvironment. Studies highlight that *CXCR4* expression is particularly elevated in lung cancer cells, closely linked to its critical roles in tumor cell proliferation, metastasis, and the formation of the tumor microenvironment [37]. High *CXCR4* expression is not only associated with tumor initiation and progression but also closely related to patient prognosis, making it clinically significant to thoroughly investigate its expression patterns and regulatory mechanisms. *CXCR4* expression is regulated at multiple levels, including transcriptional regulation, post-transcriptional modifications, and post-translational modifications. Together, these multi-level regulatory mechanisms determine the functional changes in *CXCR4* under various physiological and pathological conditions.

### 3.1. Regulation of CXCR4 Transcription Levels

Transcription factors directly control *CXCR4* transcription levels by binding to its gene promoter region. For example, *c-Myb* has been shown to regulate *CXCR4* expression, with at least 10 potential *c-Myb* binding sites identified in the *CXCR4* promoter region and regulated by Myb protein [38]. Another study found that *SP1* is a frequently binding transcription factor in the core promoter region of *CXCR4*, with evidence showing that *miR-1*35a can inhibit *CXCR4* expression by targeting *SP1* [39]. Quintana AM’s team also noted *SP1* binding sites in the *CXCR4* promoter region and suggested that *SP1* may promote *CXCR4* transcription by binding to the *CXCR4* promoter [40]. Additionally, *NF-κB* can bind to the *CXCR4* promoter region, boosting its transcriptional activity and substantially increasing *CXCR4* expression, with this regulatory effect being particularly pronounced in inflammatory and tumor cells [7]. *NF-κB* can also promote cancer cell migration and invasion through the *CXCL12*/*CXCR4* axis [41]. Furthermore, under hypoxic conditions in vitro, *HIF-1α* can bind to the *CXCR4* promoter region, significantly upregulating *CXCR4* expression and thereby enhancing cellular migration capacity [42,43].

Notably, inflammatory signaling pathways form a positive feedback loop to regulate *CXCR4* transcription. For instance, in in vitro glioblastoma cell models, TNF-α stimulation increases *NF-κB* binding to the *CXCR4* promoter, leading to upregulation of *CXCR4* expression—a mechanism also validated in in vitro NSCLC cell lines [44]. Moreover, downstream signaling pathways activated by *CXCR4* can feedback-regulate its transcription: sustained activation of the *PI3K*-*AKT* pathway promotes *CXCR4* gene transcription by phosphorylating transcription factors such as *NF-κB* [45].

DNA methylation also significantly influences *CXCR4* expression as a key epigenetic modification. For example, in peripheral blood mononuclear cells from patients with chronic hepatitis B-related liver fibrosis/cirrhosis, hypomethylation of the *CXCR4* promoter region was significantly associated with increased *CXCR4* expression levels [46]; whereas in pancreatic cancer, hypermethylation of the *CXCR4* promoter was associated with low expression. In normal pancreatic tissue, the 5′ CpG island of the *CXCR4* gene is unmethylated, whereas hypermethylation of the promoter region was detected in 45% of pancreatic cancer cell lines and 46% of primary pancreatic adenocarcinomas, with a significant negative correlation between methylation and *CXCR4* mRNA expression levels [47]; Additionally, in hepatocellular carcinoma, downregulation of *UHRF1* activates the *CXCR4*/*AKT*-JNK/IL-6/*Snail* signaling axis, thereby increasing tumor malignancy [48]. Other studies have also indicated a negative correlation between DNA methylation and *CXCR4* expression, with high methylation of the *CXCR4* gene promoter region inhibiting its transcription. Analyzing the DNA methylation status of the *CXCR4* promoter region in gastric cancer cells found that high methylation was significantly associated with *CXCR4* expression downregulation [49]. These factors influencing *CXCR4* expression provide important references for the diagnosis and treatment of lung cancer.

In addition to transcription factors and epigenetic modifications, the tumor microenvironment regulates *CXCR4* transcription via paracrine mechanisms. Immune-suppressive cells such as tumor-associated macrophages (TAMs) and regulatory T cells (Tregs) secrete cytokines like *CXCL12* and IL-6, inducing tumor cells to upregulate *CXCR4* expression. Meanwhile, growth factors secreted by tumor-associated fibroblasts (CAFs), such as EGF and bFGF, enhance *CXCR4* transcriptional activity through downstream signaling pathways [50]. This microenvironment-mediated regulation tightly links *CXCR4* expression to the tumor’s immunosuppressive state and stromal remodeling processes.

Ligand-receptor interaction and co-receptor crosstalk also contribute to transcriptional regulation of *CXCR4*. As the high-affinity ligand of *CXCR4*, *CXCL12* binds specifically to *CXCR4* to trigger conformational changes in the receptor’s intracellular domain, initiating G protein coupling and signal cascades. Sustained *CXCL12* stimulation further upregulates *CXCR4* expression on tumor cell surfaces, forming a positive feedback loop that amplifies its oncogenic functions [30,51]. *CXCR7*, another functional receptor for *CXCL12*, modulates *CXCR4* activity through dual mechanisms: forming heterodimers with *CXCR4* to synergistically enhance downstream *PI3K*-*AKT* and *MAPK*/*ERK* signaling, or competitively binding *CXCL12* to regulate local ligand concentrations and indirectly affect *CXCR4*-mediated chemotaxis [30,52].

### 3.2. Post-Transcriptional Regulation

Post-transcriptional regulation of *CXCR4* plays a critical role in various cellular functions and disease processes. Recent studies have revealed that non-coding RNAs (including miRNAs and lncRNAs) significantly affect the post-transcriptional regulation of *CXCR4*, influencing its expression levels and functional activity through complex regulatory networks.

#### 3.2.1. miRNA Regulation of CXCR4

Direct targeting regulation: Studies have shown that *miR-1*25b [53], *miR-1*46a [51], *miR-1*55 [54], and *miR-21* [55] can inhibit *CXCR4* translation by directly binding to its mRNA 3′UTR region. For instance, *miR-1*46a is induced by the LMP1 protein in EB virus-infected cells. LMP1, an EB virus-encoded protein, activates multiple intracellular signaling pathways, including the *NF-κB* signaling pathway, thereby inducing the expression of *miR-1*46a. *miR-1*46a then binds to the 3′UTR region of *CXCR4* mRNA, inhibiting *CXCR4* translation, forming an LMP1-*miR-1*46a-*CXCR4* regulatory axis, ultimately affecting cellular proliferation and metastasis capacity [53,56]. In breast cancer cells, *miR-1*46 can directly target *CXCR4*, inhibiting its expression, suppressing *NF-κB* activity, and reducing the cells’ metastatic potential. In colorectal cancer, *miR-1*25b, induced by the *CXCL12*/*CXCR4* axis, can bind to *CXCR4* mRNA 3′UTR, enhancing autophagy, thereby promoting the invasion of colorectal cancer cells while conferring resistance to 5-fluorouracil. Furthermore, *miR-9a-5p* can directly target and downregulate *CXCR4* expression, thus inhibiting *MAPK* pathway activation and alleviating lung injury in rats [57,58].

Notably, miRNA-mediated regulation of *CXCR4* exhibits tumor-specific and functional diversity. In colorectal cancer, *miR-1*26 expression is significantly reduced, and it directly targets the 3′UTR of *CXCR4* to suppress its expression, thereby blocking the RhoA signaling pathway and reducing tumor cell migration, invasion, and epithelial–mesenchymal transition (EMT) processes; patients with low *miR-1*26 expression have poorer prognosis [59,60,61]. In hepatocellular carcinoma, activation of the *CXCL12*/*CXCR4* axis induces *miR-1*25b expression, which in turn enhances *CXCR4* activity, forming a positive feedback loop that promotes tumor stemness and resistance to 5-fluorouracil (5-FU) chemotherapy [51]. Additionally, *miR-372* expression is downregulated in hepatocellular carcinoma tissues, and it directly targets *CXCR4* mRNA to inhibit cell proliferation, EMT, and anti-apoptotic capacity [62]. *miR-9a-5p* also directly targets the *CXCR4* 3′UTR, reducing *MAPK*/*ERK* pathway activity by 42% [44]. Moreover, *miR-1*26-3p/*miR-221-3p* inhibits *PIK3R2*/*PTEN*, thereby blocking the *CXCR4*/*AKT* signaling axis with an inhibition efficiency of 68% [44].

Indirect pathway regulation: Recent studies highlight that in macrophages, the absence of *miR-301a* can diminish cell migration and phagocytic capacity through the YY1/*CXCR4* pathway [63]. In colorectal cancer, *miR-1*269a targets *PCDHGA9*, activating *CXCR4* and *β-catenin* signaling pathways, promoting tumor cell invasion and metastasis [64]. *miR-1*910-5p influences angiogenesis by regulating *CXCR4* function through *MMRN2*, thereby affecting vascular permeability [65]. *miR-1*910-5p regulates vascular permeability via the *MMRN2*/*CXCR4* pathway; additionally, studies have shown that *miR-9a-5p* alleviates lung injury through the *CXCR4*/*MAPK* pathway.

#### 3.2.2. Long Non-Coding RNAs Participate in CXCR4 Regulation Through the Formation of Complex ceRNA Networks

Liang et al. found that *CXCR4* is upregulated in lung cancer cells, with *LINC00922* promoting lung cancer progression through the miR-204/*CXCR4* signaling axis. Experimental evidence shows that overexpression of *LINC00922* significantly upregulates *CXCR4* expression levels and enhances the migration and invasion capabilities of lung cancer cells. Clinical correlation analysis indicates that this mechanism is associated with poor patient outcomes [66].

An interesting finding is that lncRNA *NORAD* exhibits cell-type-specific regulation of *CXCR4*. In the NSCLC cell line A549 cells, lncRNA *NORAD* can downregulate *CXCR4* levels, significantly inhibiting cell proliferation, migration, and invasion [67]. However, this regulatory mechanism is controversial, as other studies report minimal basal *CXCR4* expression in A549 cells, yet *NORAD* overexpression enhances tumor spheroid formation, proliferation, and EMT progression, suggesting *CXCR4* fosters a more invasive tumor phenotype [68]. These seemingly contradictory findings may reflect the complexity of the *CXCR4* regulatory network, suggesting that its function may be highly dependent on the cellular microenvironment or specific pathological states. Further investigation into the dynamic regulatory mechanisms of *CXCR4* in different cell types and disease progression could not only clarify its dual role in tumorigenesis but also provide new theoretical basis and therapeutic strategies for targeted interventions.

Additional lncRNAs and circular RNAs (circRNAs) have been identified as key regulators of *CXCR4* through ceRNA networks. In hepatocellular carcinoma, high expression of lncRNA *HULC* specifically binds to *miR-372*, releasing its targeted suppression of *CXCR4* and thereby promoting cell proliferation, EMT, and anti-apoptotic processes [62]. In breast cancer, loss of lncRNA *MEG3* is common; *MEG3* directly binds to the chromatin regulator *CTCF*, preventing *CTCF* from binding to the *CXCR4* promoter and inhibiting its transcription. Overexpression of *MEG3* significantly reduces breast cancer cell migration [69]. Moreover, hormonal regulation via the ERβ/*circ-TMX4*/miR-622 pathway upregulates *CXCR4* expression through epigenetic modifications: *circ-TMX4* acts as a sponge for miR-622, alleviating its inhibitory effect on *CXCR4* mRNA and enhancing lung cancer cell invasion capacity by 2.3-fold [70,71,72]. Additionally, *CXCR4*-modified mesenchymal stem cell-derived exosomes can carry miR-320, which suppresses *VEGF*/*IGF-1* signaling to regulate pathological angiogenesis in diabetes-associated tumors, indirectly influencing *CXCR4*-mediated angiogenic functions [50,73].

These findings reveal the high complexity of post-transcriptional regulation of *CXCR4*: (1) regulatory mechanisms exhibit cell type and disease specificity; (2) they form multi-layered regulatory networks, including miRNA-lncRNA interactions; (3) they engage in cross-talk with multiple signaling pathways, such as *MAPK* and Wnt/*β-catenin*. Elucidating these regulatory mechanisms provides new targets for precision therapy in related diseases, particularly those involving *CXCR4* overactivation, such as tumors and inflammatory conditions. Future research should further investigate the synergistic interactions among different regulatory elements and their dynamic changes under various pathological conditions.

### 3.3. Post-Translational Modification

Post-translational modification of *CXCR4* mainly includes multiple processes such as phosphorylation, glycosylation, and ubiquitination. Regarding phosphorylation, studies have shown that the lack of phosphorylation hinders ligand-induced receptor endocytosis and cytoskeletal rearrangement, but does not significantly affect cell adhesion and chemotaxis [74]. *CXCR4* can be phosphorylated by multiple kinases, affecting its coupling with G proteins and the activation of downstream signaling pathways [75]. Structural studies have also indicated that phosphorylation of serine/threonine residues in the intracellular loop and carboxy-terminal region of *CXCR4* can regulate the recruitment of β-arrestin and signal transduction [76]. Additionally, protein kinase C (PKC) phosphorylates the Ser-324/5 site of *CXCR4*, matching PKC’s phosphorylation recognition sequence, activated post-*CXCL12* stimulation, regulating *CXCR4* endocytosis and degradation. G protein-coupled receptor kinase 6 (GRK6) plays a crucial role in *CXCR4* phosphorylation at multiple sites. Under *CXCL12* stimulation, GRK6 rapidly phosphorylates the Ser-324/5 site on *CXCR4*, delays Ser-330 phosphorylation, and rapidly phosphorylates the Ser-339 site, regulating *CXCR4* signaling [77]. Meanwhile, GRK2 and GRK3 also participate in the phosphorylation process of *CXCR4*, phosphorylating the Ser-346/347 sites of *CXCR4* and affecting the coupling of *CXCR4* with G proteins, thereby regulating signal transduction such as calcium ion release [77]. These studies underscore the complexity and dynamic nature of *CXCR4* phosphorylation, involving multiple kinases’ cooperation to finely regulate its signal transduction and cellular functions by phosphorylating different *CXCR4* sites [74].

Ubiquitination modification significantly impacts *CXCR4* function. Studies indicate that *CXCR4* undergoes K63- and K48-specific ubiquitination. Inhibition of its ubiquitination reduces protein stability, cell surface expression, and signal transduction capacity, thereby restricting cell migration [78]. The E3 ubiquitin ligase β-TrCP1 regulates *CXCR4* stability, modulating *CXCR4*-dependent signaling and HIV-1 entry. Interaction between β-TrCP1 and *CXCR4* leads to decreased *CXCR4* protein levels [79]. Freedman NJ et al. [80] found that β-arrestin-2 regulates *CXCR4* ubiquitination and degradation, impacting its cell surface expression.

Glycosylation modifications also hold significance. Absence of glycosylation in *CXCR4* reduces receptor stability, lowers its surface expression, and affects ligand binding and signal transduction [81]. Further research indicates that N-glycosylation (a ubiquitous post-translational modification process of proteins) is crucial for maintaining *CXCR4*’s normal folding, stability, and surface expression, with glycosylation site mutations impairing its function [82].

*CXCR4* also undergos acetylation, influencing its G protein binding and downstream signaling pathway activation, thereby affecting cellular chemotaxis and migration [83]. Research suggests Sirt1-dependent *CXCR4* acetylation impacts *CXCR4* co-receptor activity [84]. Additionally, *CXCR4* nitrosylation alters its conformation, affecting ligand binding and signal transduction, thereby regulating cellular immune responses and HIV-1 invasion [85].

Notably, downstream signaling pathways of *CXCR4* also regulate its function through post-translational modifications. The *MAPK*/*ERK* pathway enhances *CXCR4* protein stability by regulating post-translational modifications, reducing its degradation [45]. Furthermore, during *CXCR4*-mediated EMT, activation of transcription factors such as *Snail* and *Twist* can conversely upregulate *CXCR4* expression, forming a “signaling pathway—transcription factor—*CXCR4*” feedback loop that sustains the malignant phenotype of tumor cells [50].

**Figure 2:**
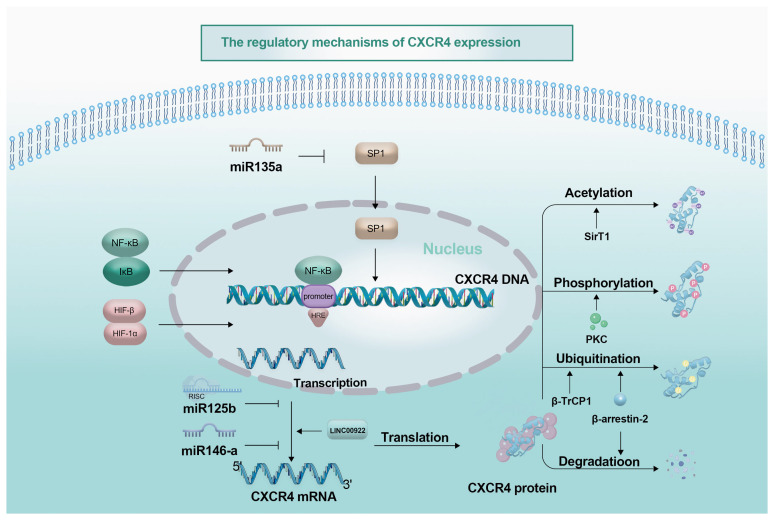
Regulatory mechanisms of *CXCR4* expression: The regulatory mechanisms of *CXCR4* expression are primarily divided into two major parts: transcriptional regulation and post-transcriptional regulation. Transcriptional regulation mainly involves transcription factors binding to the promoter region of the *CXCR4* gene to directly regulate its transcriptional level, as well as the influence of DNA methylation. In post-transcriptional regulation, the direct and indirect regulation of *CXCR4* by microRNAs (miRNAs) is primarily involved; phosphorylation of *CXCR4* affects its coupling with G proteins and activation of downstream signaling pathways; β-arrestin-2 can regulate the ubiquitination and degradation process of *CXCR4*. Specific details are described in the main text.

*CXCR4* expression is regulated at multiple levels, including transcriptional, post-transcriptional, and post-translational modifications (Figure 2). These regulatory mechanisms collectively influence the onset, progression, and outcome of tumors. In-depth investigation of the expression patterns and regulatory mechanisms of *CXCR4* not only helps elucidate its role in tumors such as lung cancer but also offers potential opportunities for developing new targeted therapeutic strategies. Targeting *CXCR4* and associated regulatory factors (such as miRNAs and lncRNAs) enables the design of precise treatments to inhibit tumor cell migration, invasion, and metastasis, improving patient outcomes.

## 4. The Dual Role of CXCR4 in Lung Cancer Progression and the Tumor Microenvironment

### 4.1. Significant Heterogeneity in the Relationship Between CXCR4 Expression and Clinical Prognosis

As an important member of the chemokine receptor family, *CXCR4* plays multiple biological roles in the development and progression of malignant tumors. Studies have shown that *CXCR4* exhibits significantly high expression in both primary and metastatic lesions of NSCLC [86]. This expression pattern is closely associated with various clinical and pathological characteristics of lung cancer. Notably, *CXCR4* expression varies significantly between histological subtypes. Meta-analysis data reveal that the positive expression rate of *CXCR4* in lung adenocarcinoma is 51.8% (337/650), compared to 50.3% (224/445) in lung squamous cell carcinoma, with a statistically significant difference between the two. Interestingly, other studies have found an even stronger association of *CXCR4* expression with lung squamous cell carcinoma.

As an important biomarker for NSCLC, *CXCR4* expression levels and their relationship with clinical prognosis have become major research foci. Extensive clinical evidence indicates that the overexpression of *CXCR4* in NSCLC tissue is significantly positively correlated with early tumor metastasis and poor clinical outcomes. However, there remains some heterogeneity in the assessment of the prognostic value of *CXCR4* across different studies. Based on existing research data, multiple meta-analysis results indicate that high *CXCR4* expression is an independent risk factor for poor prognosis in NSCLC patients. Notably, histological subtype analyses have identified significant gene mutation differences between patients with high and low *CXCR4* expression in lung squamous cell carcinoma and lung adenocarcinoma [24]. Additionally, *CXCR4* expression levels exhibit distinct specificity concerning histological subtypes. Zhu et al. found that in the specific pathological subtype of adenocarcinoma–squamous cell carcinoma (ASC), high *CXCR4* expression is significantly associated with aggressive clinical features such as lymph node metastasis (pN^+^) and advanced TNM staging (stages III-IV). Moreover, patients with high *CXCR4* expression have significantly lower 5-year disease-free survival (DFS) and overall survival (OS) rates, findings that remain statistically significant in multivariate Cox regression analysis [24,57,87].

Otsuka’s pioneering research in 2011 found that *CXCR4* was overexpressed in 78% of NSCLC tissues and significantly associated with shorter overall survival in stage IV patients (HR = 1.89, *p* < 0.01), with the effect being more pronounced in male patients [88]. However, Fung’s recent research presents a different perspective: Although *CXCR4* expression levels increase with disease progression (Stage I: 1729 ± 1083 vs. Stage IV: 2640 ± 1541), it is relatively low in early-stage patients, with no significant association between *CXCR4* expression and recurrence-free survival (*p* = 0.60) or overall survival (*p* = 0.30), and no observed gender differences. Notably, a co-expression pattern of *CXCR4* in both the nucleus and cell membrane was observed in advanced-stage patients [89], aligning with findings from Qiu’s team [90]. These findings further confirm that the *CXCR4* signaling pathway is a key molecular mechanism mediating tumor cell–matrix interactions and promoting tumor growth and metastasis.

### 4.2. Multiple Mechanisms by Which CXCR4 Promotes Tumor Progression

*CXCR4* is critically involved in numerous pathways that facilitate tumor progression, acting through a diverse array of mechanisms.

#### 4.2.1. Epithelial–Mesenchymal Transition (EMT) and Tumor Progression

One of the key processes promoted by *CXCR4* is epithelial–mesenchymal transition (EMT), a pivotal event in cancer metastasis. *CXCR4* orchestrates EMT through dual mechanisms: classical signaling pathways and autocrine loops. In the classical pathway, the activation of the *CXCL12*/*CXCR4*/*β-catenin*/*PPARδ* axis leads to an increase in *β-catenin* nuclear translocation, significantly inducing changes in EMT marker expression (upregulation of N-cadherin and downregulation of E-cadherin) [91]. These changes facilitate the cellular transitions necessary for enhanced metastatic potential.

In addition to these pathways, a novel autocrine signaling loop has been identified in *CXCR4*-overexpressing lung cancer A549 cells. Here, the Macrophage Migration Inhibitory Factor (*MIF*), a less typical ligand for *CXCR4* compared to *CXCL12*, is secreted at levels 4.5 times higher, establishing an *MIF*-*CXCR4* positive feedback loop. This loop dramatically enhances the tumor’s spheroid formation capacity [92]. Intriguingly, experimental interventions that disrupt this feedback mechanism can reverse EMT and curtail cell migration [93], underscoring this loop’s therapeutic potential.

#### 4.2.2. CXCR4 and the Immunosuppressive Tumor Microenvironment

Beyond promoting EMT, *CXCR4* plays a key role in shaping the immunosuppressive microenvironment. In lung adenocarcinoma, *CXCR4* expression is correlated with a complex immune landscape: it exhibits a moderate negative correlation with tumor purity and a moderate positive correlation with the infiltration of various immune cells, including B cells, CD4^+^ and CD8^+^ T cells, macrophages, and dendritic cells. This distinct expression pattern implies that *CXCR4* is integral to the crosstalk between tumors and the immune system, impacting tumor-immune interactions significantly. Furthermore, *CXCR4*’s expression level is of substantial clinical prognostic value.

In NSCLC patients, high *CXCR4* expression on tumor cells is strongly associated with poor prognosis and high response rates to immunotherapy [57,58,94,95,96,97]. Studies have shown that in mouse transplanted tumor models in vivo *CXCR4*^+^ cells promote myeloid-derived suppressor cell (MDSC) recruitment, suppress cytotoxic T cell function, and activate Treg cells. This orchestrated action contributes to the establishment of an immunosuppressive milieu [98,99].

The results demonstrate that within the *CXCR4*-high expressing subpopulation of MICs, cells concurrently exhibit high *PD-L1* expression, suggesting a significant positive correlation between high *CXCR4* expression status and high *PD-L1* expression. Notably, despite the concomitant high *PD-L1* expression (generally considered to mediate immune evasion), this subpopulation exhibits markedly enhanced sensitivity to immunotherapy, resulting in a 3.2-fold increase in immunotherapy response rate [57]. This observation has spurred the development of targeted therapeutic strategies. For instance, *CXCL12* inhibitors have been shown to enhance T cell infiltration in mouse transplanted tumor models in vivo. Moreover, when these inhibitors are combined with PD-1 blockers, there is a notable extension in survival in mouse models, showcasing promising translational potential [100,101].

### 4.3. Small Cell Lung Cancer

*CXCR4* can mediate the migration and adhesion of SCLC cells to mesenchymal cells. Some studies have found that it is precisely due to the migration of cancer cells to mesenchymal cells mediated by *CXCR4* and integrin that cancer cells escape necrosis caused by chemotherapy [102]. The team found through Kaplan–Meier survival analysis that the level of *CXCR4* expression significantly affects patients’ DFS (*p* = 0.005). with patients in the high *CXCR4* expression group exhibiting reduced DFS. A study used tissue microarray technology and immunohistochemistry to detect *CXCR4* protein expression in 110 lung cancer tissues and 12 corresponding normal lung tissues. The results showed that *CXCR4* protein was primarily expressed on the cell membrane and in the cytoplasm, with positive expression rates increasing in corresponding normal lung tissue, lung adenocarcinoma, lung squamous cell carcinoma, and small cell lung cancer, respectively, at 8.3%, 87.1%, 65.2%, and 100%. Additionally, *CXCR4* protein expression was closely associated with lung cancer tissue type and the invasive and metastatic capacity of lung cancer tissue [57]. Additionally, studies have reported that the *CXCR4*/*FOXM1*/*RRM2* axis is regulated by *miR-1*, thereby inhibiting the growth and metastasis of SCLC [103].

Multiple studies have demonstrated that cancer cells with high *CXCR4* expression exhibit increased tumor cell growth, invasion, and metastasis. In summary, increased *CXCR4* expression indicates poor prognosis across various cancer types. The *CXCR4* receptor, which uses chemokine *CXCL12* as its ligand, is overexpressed in SCLC patient samples and serves as the primary chemokine receptor in primary SCLC cells [104].

When chemotherapy is combined with *CXCR4* inhibitors, it reduces tumor growth to levels comparable to those achieved with combination chemotherapy alone. Combination therapy with chemotherapy and *CXCR4* antagonists can reduce metastasis in SCLC. In summary, combination therapy holds significant potential for clinical application, potentially improving overall survival rates and quality of life for SCLC patients [105].

## 5. Molecular Targeted Therapy Strategies for CXCR4

As a key regulatory molecule involved in tumor progression, *CXCR4* plays a pivotal role in mediating tumor cell migration, invasion, angiogenesis, and immunosuppressive microenvironment formation, which are closely associated with tumor metastasis and poor prognosis (Section 3 has elaborated on its multi-level regulatory mechanisms). The abnormal activation of *CXCR4* and its regulatory network provides important targets for tumor targeted therapy, and precise targeting of *CXCR4* has become a promising strategy to inhibit tumor metastasis and improve clinical outcomes.

### Exploration of CXCR4-Targeted Therapeutic Strategies

Based on a deep understanding of the *CXCR4* regulatory network, various targeted therapy strategies have been developed. For example, in terms of small-molecule inhibitor regulation, Daniela Di Paolo’s team noted that co-targeting *miR-1*26-3p and *miR-221-3p* inhibits *PIK3R2* and *PTEN*, thereby blocking *AKT* and *CXCR4* signaling pathways to reduce lung cancer growth and metastasis [94]. Similarly, *miR-9a-5p* targets *CXCR4* to inhibit *MAPK*/*ERK* pathway activation, alleviating lung injury [90,106]. In natural compounds, Matteo Gallazzi’s team found that the A009 extract from Olive Mill Wastewater (OMWW), rich in soluble polyphenols, can reduce lung cancer cell lines migration in vivo through dual inhibition of *CXCR4*/*CXCL12* [107]. Clinically, lidocaine disrupts the *CXCL12*/Ca^2+^ pathway, significantly slowing NSCLC A549 cell migration [108]. Furthermore, specific *CXCR4* antagonists, such as AMD3100, can reduce metastasis by competitively binding to *CXCR4* [109].

Combination therapy strategies also show promising results. For example, gefitinib, which upregulates *CXCR4* and EMT in PC9 lung adenocarcinoma cells, relies on TGF-β1/Smad2 signaling. Blocking *CXCR4* significantly inhibits gefitinib-induced migration, increasing EMT reversal in PC9 lung adenocarcinoma cells in vitro [110]. Notably, *CXCR4* antagonists can reverse the pro-metastatic microenvironment induced by cisplatin, reducing metastatic-initiating cells and decreasing inflammatory factor release of *CCL2* and *CXCL12*, respectively [111]. In radiation therapy, the *CXCR4*/*STAT*3/Slug pathway confers radiation resistance, whereas siRNA-mediated Slug knockout increases radiation sensitivity. Notably, engineered locked dimerized *CXCL12* has been reported to exert radiosensitizing effects by targeting the *CXCR4* axis, providing a novel strategy for enhancing radiation therapy efficacy in *CXCR4*-positive tumors [112], which aligns with the critical role of the *CXCL12*/*CXCR4* axis in maintaining cancer stem cells (CSCs) and inducing tumor dissemination, while also increasing chemotherapy resistance [113]. For example, *CXCR4* influences tumor cell resistance through the *CXCR4*/*STAT*3 signaling pathway in chemotherapy resistance mediated by bone marrow stromal cells in acute myeloid leukemia [114]. Recent studies have revealed the potential mechanism by which the *CXCR4*/*STAT*3/Slug signaling pathway is involved in NSCLC cell radiation resistance. Slug has been identified as the downstream effector molecule of the *CXCR4*/*STAT*3 signaling pathway in NSCLC cell radiation resistance, and *CXCR4*-mediated *STAT*3 activation is crucial for NSCLC cell radiation resistance. Upregulation of Slug via the *CXCR4*/*STAT*3 signaling pathway and siRNA-mediated downregulation can enhance the sensitivity of NSCLC cells to ionizing radiation (IR). This suggests that the *CXCR4*/*STAT*3 signaling pathway could serve as a therapeutic target to enhance NSCLC cell sensitivity to IR, offering potential for targeting *CXCR4* signaling pathway inhibition and eliminating NSCLC tumor stem cells. Blocking the *CXCR4*/*STAT*3 pathway may be an effective method to enhance NSCLC cell sensitivity to IR, thereby providing beneficial assistance for NSCLC radiotherapy [115].

In immunotherapy, *CXCR4* exhibits a cell-type-dependent dual role Table 1. Specifically, *CXCR4* antagonists target *CXCR4* expressed on immunosuppressive cells rather than tumor cells, and their combination with PD-1 inhibitors shows positive synergistic effects: this combination increases CD8+ T cell infiltration and reduces Treg proportions in the tumor microenvironment, thereby extending survival in the MC38 model. In contrast, when the proportion of *CXCR4*+ Tregs in peripheral blood exceeds 25%, the objective response rate of immunotherapy decreases by 42%, and progression-free survival is shortened by 5.3 months—consistent with the role of *CXCR4* in mediating Treg recruitment to the tumor microenvironment and suppressing anti-tumor immunity. In terms of microenvironment remodeling, *CXCR4*-targeted therapy promotes the formation of tertiary lymphoid structures, increasing tertiary lymphoid structures (TLS) numbers by 3.5 per field of view and significantly enhancing antigen presentation efficiency. Additionally, in a human melanoma PES43 xenograft model expressing PD-1, the combination of the human-specific *CXCR4* antagonist Pep R54 with nivolumab also demonstrated tumor growth inhibition effects [116]. Further studies indicate that the combination of *CXCR4* antagonists and PD-1 blockade holds significant potential in hepatocellular carcinoma (HCC) models. Concurrent blockade of *CXCR4* and PD-1 can reprogram conventional type 1 dendritic cells (cDC1s) within tumors, thereby enhancing antitumor immune responses [117]. In breast cancer, the combination of the *CXCR4* antagonist balixafortide with anti-PD-1 therapy has demonstrated significant therapeutic potential. The combination therapy not only reduced tumor growth and prolonged overall survival but also increased the formation of TLS in the tumor microenvironment, as shown by histological analysis. Further single-cell RNA sequencing analysis indicated that this combination therapy reduced the expression of immune checkpoint genes and exhaustion markers in T cells, suggesting a overall reduction in myeloid-mediated immune suppression [118].

**Figure 3:**
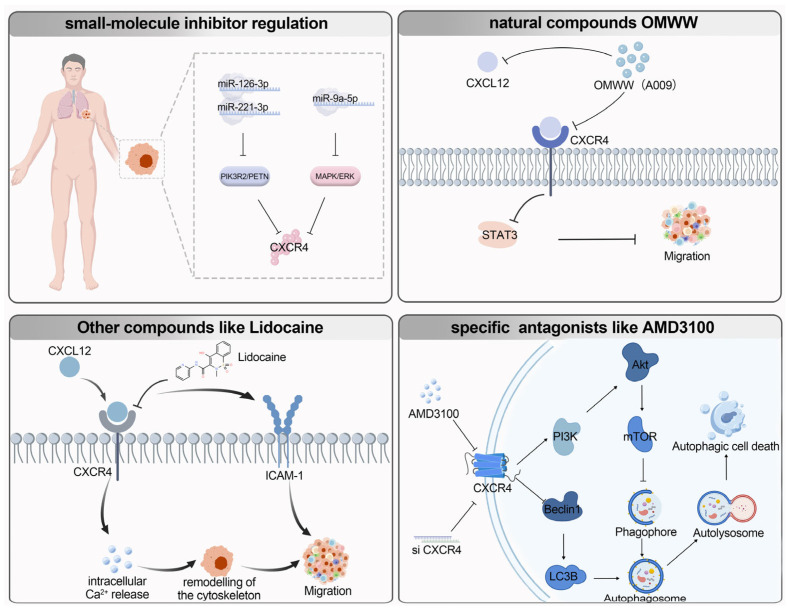
*CXCR4*: molecular mechanisms and targeted therapy in lung cancer.

In immunotherapy prediction, particularly in NSCLC studies, *CXCR4* and its interactions with the immune microenvironment have emerged as critical factors influencing the efficacy of immunotherapy. In patients with stage IA to IIB NSCLC who underwent surgery, studies have shown that *CXCL12* is expressed in most NSCLC tissue sections, and its expression levels are closely associated with the degree of tumor inflammation. Specifically, the co-existence of *CXCL12* and activated TILs CD4^+^*CXCR4*^+^CD69^+^ cells may influence tumor progression by shaping the immune cell population infiltrating lung adenocarcinoma [59]. In this context, *CXCR4* blockade has been found to potentially increase the number of tumor-infiltrating T lymphocytes and exhibit synergistic effects with immune checkpoint inhibitors. However, high *CXCR4* expression in peripheral T lymphocytes may be associated with poorer immunotherapy efficacy, suggesting that *CXCR4* plays a complex role in immunotherapy. These findings highlight the dual role of *CXCR4* in immunotherapy across different tumor types: it may enhance immunotherapy efficacy in some cases while potentially weakening it in others, depending on its specific function and expression pattern within the tumor microenvironment (Figure 3).

## 6. Outlook

In terms of disease diagnosis and treatment, the expression levels of *CXCR4* may vary across different types of tumors, potentially serving as a biomarker for disease prognosis and a potential drug target [9]. In NSCLC, *CXCR4* expression levels could serve as a novel tumor marker for predicting patient outcomes. Higher *CXCR4* expression in NSCLC has been linked to shorter overall survival (OS) [19]. In a study of 2932 lung cancer patients, high *CXCR4* expression was associated with shorter OS and disease-free survival compared to low expression. In NSCLC, poor overall survival was significantly associated with *CXCR4* expression in the cell membrane and cytoplasm. The study also showed that *CXCR4* expression is associated with male gender, advanced tumor stage, advanced nodular stage, distant metastasis, advanced TNM staging, and epidermal growth factor receptor expression. However, it is not associated with age, smoking history, histopathology, lymphatic invasion, or local recurrence [23]. However, the current detection of *CXCR4* expression lacks a standardized system, with inconsistent immunohistochemistry interpretation criteria and mRNA quantification methods employed across different studies. Therefore, the clinical applicability of *CXCR4* as a prognostic biomarker still requires validation through multicenter large-scale studies.

Given the important role of *CXCR4* in NSCLC, its genetic and structural characteristics have also attracted widespread attention. Research indicates that *CXCR4* is translated into five different splice variants of varying lengths, each with distinct amino acids at the N-terminal end. Since the N-terminal end is the first recognition site for chemokines, *CXCR4* variants may exhibit different responses to *CXCL12*. Hee-Kyung Park’s team [82] explored *CXCR4* variants expression in cell lines and found that most cell lines express more than one variant. The N-terminal sequence of each *CXCR4* variant determines receptor expression and influences ligand recognition. Functional analyses suggest that *CXCR4* variants may also interact or influence each other during *CXCL12*-stimulated cellular responses. These findings suggest that *CXCR4* variants could have unique functional roles warranting further investigation and may pave the way for new drug interventions, particularly for lung cancer. The impact of N-terminal sequence variations among *CXCR4* splice variants on ligand recognition affinity, downstream signal transduction specificity, and tumor subtype adaptability remains a critical scientific question to be urgently elucidated in this field.

Additionally, Nanako Kawaguchi’s team revealed through studies using gene-knockout mice that *CXCR4* is involved in cardiac development and brain development. Mice with homozygous *CXCR4* knockout died before birth. Thus, *CXCR4* is a key molecule for normal development. Interestingly, *CXCL12*-expressing cells are present in the outflow tract, while *CXCR4*^+^ cells are found in the neural c*REST*. Thus, *CXCR4* may be involved in the migration of neural c*REST* cells during normal development [119]. The core regulatory role of *CXCR4* in normal physiological processes provides an important reference for reverse inference of its aberrant activation mechanisms in tumors, yet the molecular regulatory network connections between the two remain to be further elucidated.

In terms of therapeutic development, several small molecules antagonizing *CXCR4* have been created [120]. The FDA-approved first *CXCR4* antagonist, plerixafor (AMD3100), is currently used to mobilize hematopoietic stem cells for the treatment of non-Hodgkin lymphoma [10,119] and has been shown not to exhibit cross-resistance with T134 (another small-molecule *CXCR4* inhibitor that binds to *CXCR4*) [121]. Similarly, MSX-122 for vascular diseases and AMD11070 for HIV treatment have completed clinical trials [10]. A C-terminally modified small cyclopeptide, LY2510924 [116], will also serve as a universal scaffold for developing *CXCR4*-targeted probes or therapeutic agents for tumor imaging or treatment [122]. Additionally, an anti-*CXCR4* antibody, LY2624587 has been developed for targeting tumors and cancers [105,118]. Additional *CXCR4* antagonists are listed in Table 2.

Current clinical application of *CXCR4*-targeted therapeutics remains challenged by side effects such as hematopoietic suppression and infection risk, necessitating formulation optimization (e.g., tumor microenvironment-responsive preparations) and adjustment of combination regimens to reduce systemic toxicity and enhance tumor targeting specificity. Meanwhile, the development and clinical translation of *CXCR4*-specific molecular imaging probes (such as 68Ga-pentixafor PET) hold promise for integrating precise patient selection with dynamic monitoring of therapeutic efficacy. Moreover, the crosstalk mechanisms between *CXCR4* and signaling pathways including *PI3K*/*AKT*, *MAPK*/*ERK*, and Wnt/*β-catenin* provide novel insights for developing multi-target combination therapeutic strategies, which will require validation of their efficacy and safety through additional preclinical and early-phase clinical studies in the future.

## 7. Conclusions

*CXCR4* has emerged as a promising diagnostic and therapeutic target in lung cancer due to its abnormal high expression in both NSCLC and SCLC, with significant histological subtype heterogeneity, and strong association with tumor progression. Research indicates that *CXCR4* expression correlates with the invasiveness and metastatic potential of lung cancer and directly impacts patient prognosis—especially in adenosquamous carcinoma (ASC), high *CXCR4* expression is closely associated with lymph node metastasis and advanced TNM staging, serving as an independent poor prognostic factor. As a crucial regulator of tumor cell migration and invasion, *CXCR4* may also modulate tumor immune responses by influencing immune cell function within the tumor microenvironment, such as recruiting myeloid-derived suppressor cells and activating regulatory T cells. *CXCR4* expression is regulated at transcriptional (transcription factor binding, DNA methylation), post-transcriptional (miRNA/lncRNA ceRNA network), and post-translational (phosphorylation, ubiquitination, glycosylation)multiple levels, and its expression levels can serve as a prognostic biomarker for lung cancer and represent a potential drug target. Recent research advances indicate that *CXCR4*-targeted therapy—including small-molecule antagonists, anti-*CXCR4* antibodies, and combination strategies with chemotherapy, immunotherapy, or targeted therapy—shows promising potential in enhancing treatment efficacy and reducing tumor recurrence, offering new directions for future treatment strategies. However, the exact mechanisms of *CXCR4* in lung cancer and its role as an optimal therapeutic target require further clarification.

## 8. Discussion

**Figure 4:**
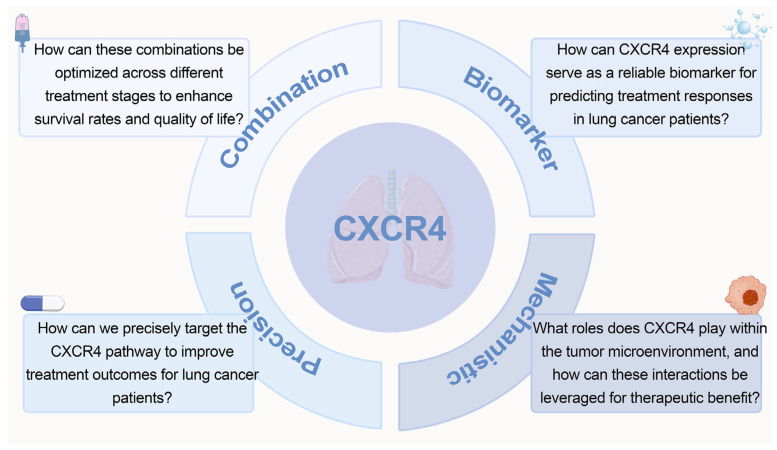
A diagram of several key scientific issues that remain to be addressed in the future.

As summarized earlier, *CXCR4* acts as a core regulatory hub in lung cancer, integrating multiple oncogenic processes including tumor cell proliferation, invasion, distant metastasis, tumor microenvironment (TME) remodeling, and chemoresistance, which collectively supports its potential as a translational therapeutic target. Despite this well-established theoretical basis, current research progress and clinical translation of *CXCR4*-targeted strategies still face multiple unresolved bottlenecks. At the basic research level, the functional specificity of *CXCR4* splice variants remains undefined—the impact of N-terminal amino acid sequence variations on ligand binding affinity and downstream signal transduction lacks systematic validation, precluding explanation of differential responses to targeted therapy among patients. Simultaneously, bidirectional regulatory mechanisms between *CXCR4* and stromal/immune cells in the tumor microenvironment remain poorly understood, particularly the regulatory threshold for tumor progression concerning the synergistic/competitive relationship between the *CXCL12*/*CXCR4* axis and *CXCR7*. At the clinical translation level, the primary issue is the lack of a standardized system for *CXCR4* expression detection, with inconsistent immunohistochemistry interpretation criteria and mRNA quantification methods across studies, limiting its clinical applicability as a prognostic biomarker. Second, current *CXCR4* antagonists exhibit side effects including hematopoietic suppression and infection risk, with insufficient tumor tissue targeting, and mature solutions for toxicity reduction through formulation optimization or combination regimens have yet to be established. Furthermore, predictive models for *CXCR4*-targeted therapy response in lung cancer patients with different pathological subtypes and genetic backgrounds (e.g., EGFR mutations, ALK fusions) remain undeveloped, and precise identification of beneficiary populations remains a critical issue requiring urgent breakthrough. Finally, research on mechanisms of *CXCR4*-mediated chemoresistance in SCLC lags behind that in NSCLC, with clinical exploration of related targeted strategies still in early stages. Future studies should advance the precision application of *CXCR4*-targeted strategies. At the basic research level, it is necessary to thoroughly investigate the functional heterogeneity of *CXCR4* splice variants and clarify their expression profiles and regulatory networks across different lung cancer subtypes. Simultaneously, through technologies such as single-cell sequencing and spatial transcriptomics, elucidate the cell-specific functions of *CXCR4* in the tumor microenvironment and reveal its synergistic regulatory mechanisms with immune checkpoint molecules. In the field of technology development, clinical translation of *CXCR4*-specific molecular probes should be promoted. Meanwhile, optimize targeted drug design and develop tumor microenvironment-responsive novel antagonists or antibody-drug conjugates (ADCs) to enhance targeting specificity and reduce systemic toxicity. At the clinical translation level, multicenter, large-scale clinical studies are needed to validate the synergistic prognostic value of *CXCR4* expression with existing clinical indicators (such as TNM staging, genetic test results) and establish standardized detection protocols. Design individualized combination therapy regimens for different lung cancer subtypes, such as combining *CXCR4* antagonists with PD-1/*PD-L1* inhibitors in NSCLC and with chemotherapy agents in SCLC. Future research should focus on the following key questions: First, how can the *CXCR4* pathway be precisely targeted to improve patient treatment responses? Second, can *CXCR4* expression serve as a reliable biomarker for predicting patient responses to specific treatments? Additionally, can the combination of *CXCR4*-targeted therapy with other treatment methods (such as immunotherapy) yield more significant therapeutic effects? The answers to these questions will have a significant impact on the application of *CXCR4* in lung cancer treatment and may provide patients with more effective treatment options. By thoroughly exploring the mechanism of action of *CXCR4* and its integration with other treatment strategies, future research holds promise for bringing new hope to lung cancer patients (Figure 4).

In summary, relying on its core regulatory role in lung cancer progression, *CXCR4* remains a highly translational therapeutic target. The success of its clinical application depends on in-depth analysis of core mechanisms, effective breakthrough of technical bottlenecks, and precise optimization of clinical protocols. In the future, through interdisciplinary collaboration, it is expected to provide new treatment options for lung cancer patients and ultimately improve patient prognosis.

## Figures and Tables

**Table 1:** The Dual Role of *CXCR4* in Immunotherapy.

Direction of Effect	Level of Action	Specific Mechanisms	Biological Effect/Clinical Significance
Positive Effects [116,117,118]	Tumor Microenvironment Level	Promotes the formation of Tertiary Lymphoid Structures (TLS); Reprograms Type 1 Conventional Dendritic Cells (cDC1s)	Increases TLS count by 3.5 per field of view, enhancing antigen presentation efficiency; Boosts T cell-mediated anti-tumor immune response
	Tumor Cell—Immune Cell Interaction Level	Used in combination with PD-1 inhibitors; Reduces the proportion of Regulatory T Cells (Tregs); Attenuates myeloid-mediated immunosuppression	Increases CD8+ cytotoxic T cell infiltration by 2.1-fold; Decreases Treg proportion from 32% to 15%, relieving immunosuppression; Extends survival by 37%, amplifying the efficacy of immunotherapy
Negative Effects [116,118]	Tumor Microenvironment Level	Over-activation of the CXCR4/CXCL12 axis; Recruits immunosuppressive cell infiltration (Tregs, MDSCs, etc.)	Strengthens the immunosuppressive microenvironment; Impairs the anti-tumor function of effector T cells
	Immune Cell Level	Aberrantly high expression of CXCR4 on T cells; Affects T cell activation, migration, and functional maintenance	Decreases the objective response rate to immunotherapy by 42%; Shortens progression-free survival by 5.3 months

**Table 2:** Overview of *CXCR4*-Related Antagonists Research.

Category	Antagonist Name	Core Data
Small-molecule antagonists	AMD3100 (Plerixafor)	Clinically approved for hematopoietic stem cell mobilization [109,123];
	AMD11070 (Mavorixafor)	The first oral *CXCR4* antagonist proven effective in Phase III clinical trials [124];
	LY2510924	Specifically blocks the *CXCR4*/*CXCL12* axis, exerting significant in vitro/in vivo anti-leukemia activity (covering AML, CLL, ALL);Exerts a synergistic effect when combined with chemotherapeutic drugs such as cytarabine and daunorubicin, enabling reduced chemotherapy dosage and enhanced efficacy [125];
	POL6326 (balixafortide)	Exhibits dose-dependent hematopoietic stem cell mobilization after subcutaneous injection [126];
	MSX-122	Particularly important in the treatment of metastatic cancer [127];
	WZ811	Characterized by rapid in vivo metabolism and low tissue distribution specificity [128];
	AMD3465	High affinity, high selectivity, low cytotoxicity, and good water solubility [129,130];
Peptide antagonists	T22	Potent *CXCR4* peptide antagonist with anti-HIV-1 and anti-tumor metastasis activities [131];
	FC131	High affinity, with anti-HIV and anti-tumor activities [132];
	EPI-X4-8mer	Smaller molecular weight and easier to synthesize [133];
	BPRCX807	Remodels the tumor microenvironment by inhibiting the *CXCR4*/*CXCL12* axis, thereby enhancing the efficacy of PD-1 inhibitors [134];
	TN14003	Low cytotoxicity and specific regulation of *miR-1*46a-5p [135];
	Motixafortide (BL-8040)	BL-8040 combined with pembrolizumab + chemotherapy (nab-Paclitaxel + Gemcitabine) for the treatment of advanced pancreatic cancer;Mobilizes hematopoietic stem cells, reduces MDSC infiltration in the tumor microenvironment, increases CD8^+^T cell infiltration, reverses the immunosuppressive microenvironment, and enhances the anti-tumor activity of PD-1 inhibitors [136];
Monoclonal antibody antagonists	BMS-936564	Phase II clinical study for multiple myeloma (NCT01837091);Combination with PD-1 inhibitors prolongs progression-free survival by 4.2 months [137];
	PF-06747143	Preclinical data confirms potent in vitro and in vivo activity against various hematological malignancies (MM, ALL, CLL), with extremely low toxicity to normal hematopoietic stem cells and good safety profile [138]

## Data Availability

No new data were created or analyzed in this study. Data sharing is not applicable to this article.

## Abbreviations

NSCLC	non-small cell lung cancer
SCLC	small cell lung cancer
*CXCR4*	C-X-C chemokine receptor 4
GPCR	G protein-coupled receptor
SDF-1	stromal cell-derived factor 1
DRY motif	Aspartate-Arginine-Tyrosine triplet
*PI3K*	Phosphoinositide 3-kinase
*AKT*	Protein kinase B
JNK	c-Jun N-terminal kinase
TME	tumor microenvironment
TCR and BCR	T cell and B cell receptors
IGS	immune gene surrender
EMT	epithelial–mesenchymal transition
LMP1	EB virus-encoded protein
*LINC00922*	Long Intergenic Non-Protein Coding RNA 00922
PKC	protein kinase C
GRK6	G protein-coupled receptor kinase 6
HIV-1	Human Immunodeficiency Virus type 1
ASC	adenocarcinoma-squamous cell carcinoma
DFS	disease-free survival
OS	overall survival rates
*MIF*	Macrophage migration inhibitory factor
MDSC	Myeloid-derived suppressor cells
MICs	metastasis-initiating cells
OMWW	Olive Mill Wastewater
CSCs	cancer stem cells
IR	ionizing radiation
TLS	tertiary lymphoid structures
HCC	hepatocellular carcinoma
cDC1s	type 1 dendritic cells
